# Combining Grey Relationship Analysis and Neural Network to Develop Attractive Automobile Booth Design

**DOI:** 10.1155/2020/8863727

**Published:** 2020-06-20

**Authors:** Xinhui Kang

**Affiliations:** School of Art and Design, Nanchang University, No. 999 Xuefu Avenue, Nanchang 330031, Jiangxi, China

## Abstract

Miryoku engineering is a design concept based on customer preferences, with the goal of creating attractive products or spaces. However, traditional Miryoku engineering faces two main issues: (1) the upper Kansei factor ranks the weights by the number of mentions, but it does not represent the importance of customers; (2) the mapping connection between the upper Kansei factor and the lower specific conditions adopts a statistical analysis method, which easily leads to the omission of key information. With the development of computer-based artificial intelligence, it repeatedly simulates human thinking with simple calculation rules, which has the advantages of fewer errors and faster speed. Therefore, on the three-level evaluation grid diagram platform established by Miryoku engineering, this paper first uses grey relationship analysis to comprehensively evaluate the priority order of Kansei words. Secondly, for the key Kansei factors, a morphological deconstruction table that connects the original reasons and specific conditions is established. Orthogonal design is used to screen representative combinations of design elements and create sample models by using the 3D software. Finally, the neural network was used to establish a mapping function between the key Kansei factors and the representative product design elements, and based on this, the most perceptually attractive product design was discovered. As a case study, the automobile booth was used to validate the effectiveness of the proposed method and significantly improve exhibitor design decisions and attendees' satisfaction.

## 1. Introduction

**Figure 1:**
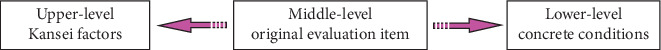
Evaluation grid diagram.

The purpose of production is to design an attractive product that meets customer preferences to increase market share. However, an attractive product is difficult to be defined by appropriate design elements [1–4]. Therefore, it is important to build a communication channel between the customer's charm preferences and product design elements. Miryoku engineering is one of the branches of Kansei engineering since 1998, when the Japan Society of Kansei Engineering (JSKE) was established [5]. It is also a type of ergonomic study of the relationship between metal perception and actual objects. Miryoku engineering is a research group initiated by a Japanese scholar Masato Ujigawa [6], bringing together a number of scholars. Its goal is to create attractive products, space technology, and knowledge, which is mainly to respond to the shift from the production-oriented to consumer-oriented concept, so that there is a smooth communication interface between designers and customers to develop products in the direction of customer needs. “Miryoku” is a Japanese word which means “power of attractiveness.” Miryoku comes from the inner self-image, which is a kind of attraction and influence that makes people feel satisfied after they have Kansei, and then the attractive perception and memory are produced [7]. The Miryoku engineering process can be used to dig out the three-level charm factors of human-to-things, namely, the upper Kansei factor, the median original reason, and the lower specific condition (Figure 1). The evaluation structure chart established through the connection can intuitively provide a qualitative reference for the designer and can also quantify the customer's fuzzy attractive needs into specific design parameters that can be understood by the company through statistical analysis.

In the recent years, many scholars have successfully applied Miryoku engineering in the fields of car form design [8], ecological tourism [9], music games [10], and other fields, such as Han and Ma [11] used the method of Miryoku engineering to capture the charm factor of the stationary store. Park et al. [12] applied Miryoku engineering to analyze the user experience design of mobile hospital applications. Shen [13] and Chen et al. [14] utilized Miryoku engineering to explore the appeal of Facebook social networking service games from the perspective of game usability and functionality. People's charm experience is affected by social background, education, and aesthetics, and Miryoku engineering can perform very specific calculations on this ambiguity. However, there are still two shortcomings in the above article: (1) the extraction of the higher representative Kansei factors is mostly based on the importance of the frequency ranking mentioned in the interview process. The number of mention times is only related to the subject's feelings and cannot be linked to the importance. There are also some scholars who use the analytic network process [15] or the analytic hierarchy process [16] to calculate and rank the weight of each attractive factor, but these methods are limited by the subjective evaluation of the subject, and it is easy to have deviations in results. (2) The linear quantification method establishes a mapping function between the upper Kansei factor and the lower specific conditions. It is difficult to accurately measure the customer's nonlinear and nonnormal emotional imagery [17]. Multiple linear regression methods (e.g., quantification theory type I, QT-I) can be used to statistically predict the relationship between a Kansei classification and the design elements. Like Ho and Hou [18], Ko et al. [19] combined Miryoku engineering and QT-I to explore the attractive factors of the APP icon and the office chair. However, the linear regression method can only evaluate the relationship between length and height of linear values, and obviously it cannot measure nonlinear emotion variables. Therefore, this paper proposes the application of grey relationship analysis (GRA) and the neural network (NN) in artificial intelligence technology to the research process of charisma to address the above problems and develops a similar product design using human logic reasoning. GRA is an important part of the grey system theory. In addition to its simplicity and accuracy, it can also convert multicriteria decision problems into quantitative analysis and comprehensive evaluation. So, GRA is used to measure the relative weight and priority of the upper evaluation items in the evaluation grid diagram. The NN can simulate the nonlinear mapping relationship with fast convergence and good local approximation effect, which solves the fuzzy and uncertain characteristics of perceptual evaluation, and makes up for the shortcomings of traditional linear methods in losing some information. Therefore, NN is used to establish the mapping function of Kansei factors and lower concrete design conditions. Based on this, through continuous trial and error, the product design combination with the highest Kansei evaluation value is obtained, providing theoretical guidance for companies and designers to reduce time to market and risks and significantly increase customer satisfaction.

According to the literature survey, there is no literature dedicated to the display of space design perspectives by Miryoku engineering. The display design is a comprehensive art centered on commodities, which uses effective resources to beautify commodities in a certain space. The design of a booth plays an important role to determinate a trade show success in attracting visitors and providing a positive business atmosphere [20–22]. Many auto brands have begun to pay more attention in participating in trade shows instead of the development of the car itself. When the design and interior decoration of the automobile booth are done as a way to attract attendees, customers will randomly choose the exhibition area that can arouse their own interest in addition to the subjective identification of the brands they want to visit. Therefore, an attractive automobile booth design can attract more people and then achieve the best-selling purpose and can also provide an important trading platform with suppliers. However, compared with other fields (such as graphics or architectural design), an automobile booth design does not have a sufficient theoretical basis to maintain display research and analysis. Nevertheless, few studies have examined trade show booth design and planning, dealing with the issue of commercial information transmission and reception [22]. Therefore, this paper takes the automobile booth design as a case study and combines GRA and NN to develop an attractive automobile booth design during the Miryoku engineering operation stage. In addition, a brief comparison to explain what are the similarities and differences between the research in this article and the previous ones is presented in Table 1. The main contributions of this paper can be summarized as follows:

- There is no study about integrating GRA and NN into Miryoku engineering process
- Investigated the charm factors of the automobile booth design and derived attractive product forms
- GRA measured the weight and priority of the upper abstract Kansei factor
- NN replaced traditional linear analysis methods to establish a mapping relationship between Kansei factors and design languages

The organization of this paper is as follows.  Section 2 reviews the previous relative works that are based on Miryoku engineering and discusses the application of GRA and NN.  Section 3 briefly describes our proposed framework and depicts each step of the conducted research for the automobile booth design.  Section 4 presents the analysis and discussion of the research, and Section 5 concludes this paper from the investigation.

## 2. Relative Work

### 2.1. Miryoku Engineering

Evaluation grid method (EGM) is an important research method in Miryoku engineering. It is also a qualitative interviewing method to obtain the evaluation elements of attraction. Sanui and Inui [28] improved the psychological concepts of the repertory grid method proposed by Kelly and added two steps. Firstly, the subjects were asked to answer what they liked or disliked after comparing the samples in pairs. Then, they were asked the reasons for the favorite by additional questions, according to the meaning or conditions of the clarification of the problem to divide the hierarchy. EGM is helpful for deep understanding of the subject's psychological cognition level. Even abstract psychological feelings and subtle emotional changes that are difficult to capture can be extracted by this method. Liu et al. [29] used EGM of Miryoku engineering and fuzzy QFD to explore the design elements of a typical culture and creative product. Yamagishi et al. [30] carried out EGM of Miryoku engineering to increase the robustness of the Kansei quality and maximize customer satisfaction for consumer electronic products. Conducting qualitative EGM interviews with high-involvement groups can record and summarize the semantic structure of expert logic from the actual behavior cases of users through pairwise comparison of various features. The specific EGM operation process is briefly described as follows:

1. Prepare relevant questions and pictures for the interview.
2. Start a one-to-one interview and ask each respondent to divide the picture into two categories of like and dislike.
3. Delete pictures classified as dislikes.
4. Subjects are asked to briefly describe their favorite reasons in their own language to establish the respondents' original evaluation items or middle evaluation items.
5. According to the original evaluation items, ask further about the abstract reasons and the specific favorite details. Then, form upper and lower evaluation items.
6. All evaluation items are compiled into a diagram. Integrating contents of all subjects, all items are connected with straight lines to indicate the hierarchical relationships.
7. Because there are too many high-level charm factors, this study classifies those with similar content and nature according to the KJ method and assigns a name to the group. Repeat this step until you can no longer group. Through this clustering process, clear charm factors can be obtained.

### 2.2. Grey Relationship Analysis

GRA is a measurement method to analyze the correlation between discrete sequences in the grey system theory. In incomplete information, certain data processing is used to find the correlation of data between random factor sequences. Its main purpose is to quantify the degree of correlation between factors that affect the research theme [31]. In the process of developing a product design, GRA can be used to determine the structure of the design problem and the policy of design development. Wei [32] utilized GRA based on the Kansei engineering to analyze the design of modeling and color of guide signs in the public space. Chou [33] applied GRA to determine the techniques for improving the Chinese guanxi quality of ocean freight forwarders in Taiwan. Wang [34] and Wang [35] used grey system theory to describe the weighting influence of product elements on images and words and established the transparent relationships between design elements and page images. Chen and Chuang [36] combined GRA with the Taguchi method to optimize subjective quality of customer satisfaction.

The key and difficult point of Miryoku engineering is to determine the importance of ranking the upper Kansei factors in the evaluation grid diagram. The subjective method is used to obtain the ranking of the Kansei factor based on the total number of customer mentions, but the number of mentions can only represent the degree of relevance to the target product. In response to this problem, this article introduces the grey relationship degree in the GRA into Miryoku engineering. Based on the semantic evaluation of each Kansei factor, a Kansei factor is randomly set as the comparison sequence, and other Kansei factors are used as the reference sequence. Kansei factors' weighting is finally determined. GRA is mainly divided into the following six calculation steps:

Step 1: determine the comparison and reference sequences:
Set *X*_*o*_=(*x*_*oj*_|*j*=1,2, ..., *n*) as a reference sequence, also known as the parent sequence
Set *X*_*i*_=(*x*_*ij*_|*j*=1,2, ..., *n*) as a comparison sequence, also known as the subsequence *i*=1,2, ..., *m*
Step 2: the data preprocessing method includes
(1) Measuring the effectiveness of the lower-better
  (1) $$\begin{array}{c} x^{*ij}=\frac{x_{ij}-\min_{i}x_{ij}}{\max_{i}x_{ij}-\min_{i}x_{ij}}. \end{array}$$

(2) Measuring the effectiveness of the higher-better

(2) $$\begin{array}{c} x^{*ij}=\frac{\max_{i}x_{ij}-x_{ij}}{\max_{i}x_{ij}-\min_{i}x_{ij}}. \end{array}$$

(3) Measuring the effectiveness of the nominal-better

(3) $$\begin{array}{c} x_{ij}^{*}=1-\frac{\left|x_{ij}-x_{\text{obj}}\right|}{\max\left\{\max_{i}x_{ij}-x_{\text{obj}},x_{\text{obj}}-\min_{i}x_{ij}\right\}}. \end{array}$$

  Step 3: find the different sequence between the reference sequence and the comparison sequence:

(4) $$\begin{array}{c} \Delta_{oij}=\left|x_{oj}^{*}-x_{ij}^{*}\right|,\;i=1,2,\ldots ,m,j=1,2,\ldots ,n. \end{array}$$

(4) Step 4: calculate the grey relationship coefficient:

(5) $$\begin{array}{c} \gamma_{oij}=\frac{\min_{\forall j}\min_{\forall j}\Delta_{oij}+\zeta \max_{\forall j}\max_{\forall j}\Delta_{oij}}{\Delta_{oij}+\zeta \max_{\forall j}\max_{\forall j}\Delta_{oij}}, \end{array}$$

  where *ζ* is the resolution factor, which is generally taken as *ζ*=0.5 [36, 37].
  Step 5: calculate the grey relationship degree:

(6) $$\begin{array}{c} \Gamma_{oi}=\sum_{j=1}^{n}w_{j}\times \gamma_{oij},\;w_{j}\text{is the weight,}\sum_{j=1}^{n}w_{j}=1. \end{array}$$

  Step 6: arrange the grey relationship degree from small to large.

The calculated grey relationship degree is a relative weighted value. When the value is large, it indicates that the Kansei factors are more important and the designer should focus on it. On the contrary, the smaller the value is, the less important the Kansei factors are, which can be temporarily considered as an unimportant reference under the consideration of cost.

### 2.3. Neural Network

Neural network is an important branch of artificial intelligence. It is an information processing system that mimics the biological neural network and was created by biological inspiration from [38]. The backpropagation learning algorithm is one of the most widely used algorithms in NN models. It acquires external knowledge through learning and stores it in the network, which can solve difficult problems that are not easily handled by computers, especially language and image recognition, combined optimization calculations, and intelligent control. The feed-forward multilayer NN trained by the general BP algorithm consists of one input layer, one output layer, and one or more hidden layers. During the backpropagation process, the gradient steepest descent method is used to constantly adjust the weights and thresholds of the network to minimize the sum of squared errors of the network. Guo et al. [39] integrated NN and the genetic algorithm to achieve multiobjective optimization of the tricolor product color design. Wang et al. [40] constructed three NNs optimized by the genetic algorithm to predict the calculated scores of three Kansei adjectives. Misaka and Aoyama [41] applied NN to develop a design system for crack patterns on the cup surface based on Kansei. Wang et al. [42] investigated the relationships among the sneakers and the top 10 point guards by using Kansei perception and NN learning algorithms. NN can use human's nonlinear thinking methods to deal with subjective and imprecise emotional activities, so this paper uses this advantage to establish a mapping relationship between the customer Kansei charm factor and product design conditions.

## 3. Proposed Research Framework

**Figure 2:**
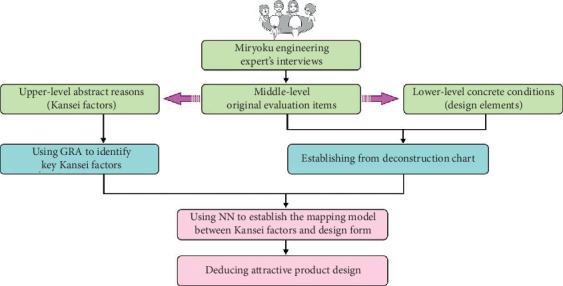
The proposed research framework.

In this study, the advantages of Miryoku engineering, GRA, and NN are combined to develop an attractive product design. The proposed research framework is shown in Figure 2. The study is divided into three phases: First, the expert group goes through the interview process of Miryoku engineering for a 3-level evaluation grid chart that captures high-level abstract reasons, median original evaluation items, and low-level specific design conditions. After that, GRA was used to identify the key upper Kansei factors and establish the corresponding morphological deconstruction chart for the Kansei factors with the highest grey correlation. Finally, NN is used to establish a mapping model between the key Kansei factor and the specific form of the product, and the attractive design of the automobile exhibition booth is derived based on this.

### 3.1. Miryoku Engineering Process

To obtain qualitative reference information, 12 high-involvement expert groups were recruited for experimental interviews. They were six males and six females, aged between 30 and 55, and all had more than five years of industrial design experience. First, 100 models of the automobile booth design images were collected from the Internet, magazines, and other channels. After removing pixel blurring and images with large environmental impact (such as light, environmental reflections, and shadows), a total of 85 cards with a size of 10 cm × 10 cm were produced. Then, the experts were invited to compare automobile booth cards with each other, and interviews were conducted from the viewpoints of pros and cons and likes and dislikes and the original reasons for preferences were refined. The time of each interview is about 50–60 minutes, and the specific questioning method is stated in the Appendix.

**Figure 3:**
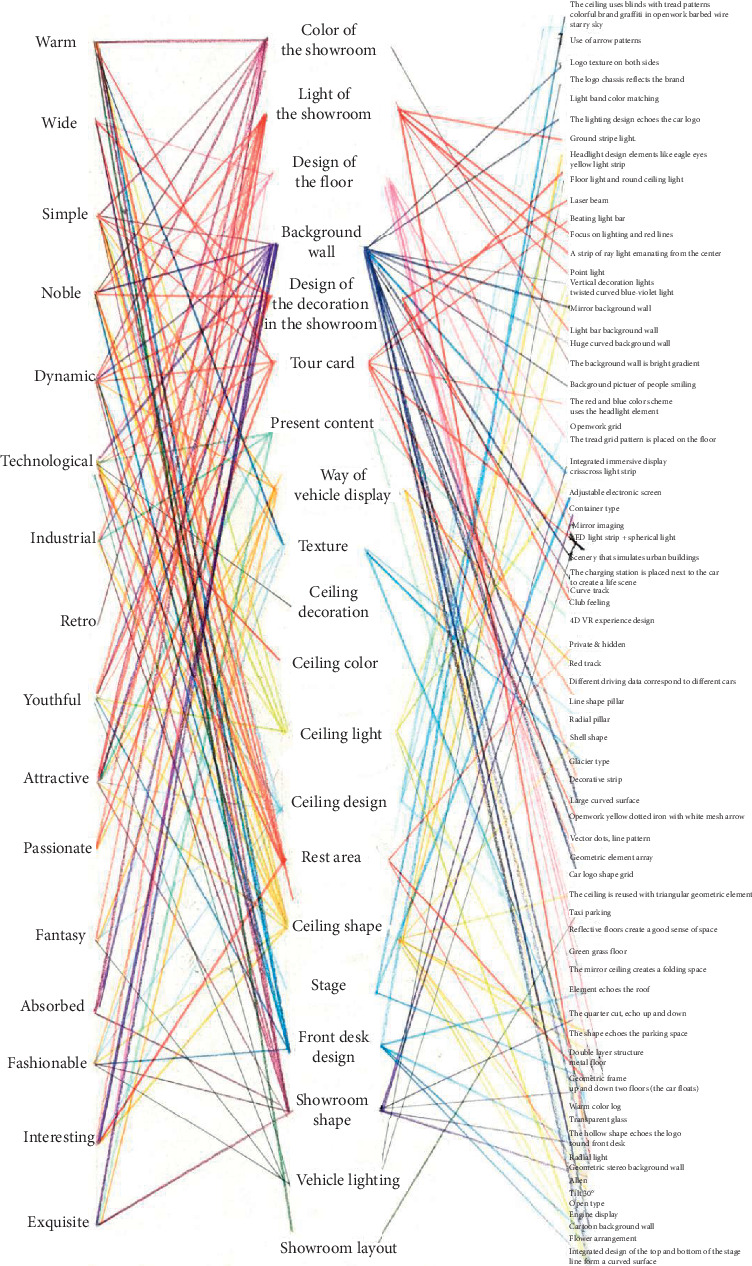
Complete evaluation structure chart.

The above steps were repeated to complete the Miryoku engineering interview with 12 experts. A total of 174 upper Kansei factors, 20 median original reasons, and 77 lower specific conditions were extracted (Figure 3). To avoid too many Kansei factors from burdening the designer, the KJ method was used to summarize the extracted upper Kansei factors, and 16 representative perceptual words (Table 2) are summarized; a complete set was built based on them and the figure of the evaluation structure was established.

### 3.2. Using GRA to Identify Key Kansei Factors

This stage uses GRA to determine the weighting of each emotional vocabulary, allowing designers to gather key Kansei factors. First of all, 14 representative samples were selected from 85 automobile booth cards, and after matching with 16 perceptual factors, 100 field experts (50 men and 50 women, aged 25–55, with more than five years of design experience) were invited. A perceptual evaluation was performed, and the average evaluation values are obtained as shown in Table 3. Secondly, the “exquisite” vocabulary is set as the reference sequence and the other 15 perceptual words as the comparison sequence. Equations (1)–(6) are used to calculate the grey relationship degree (GRD) of the 15 perceptual factors. The weight values are arranged from big to small, as shown in Table 4.

**Figure 4:**
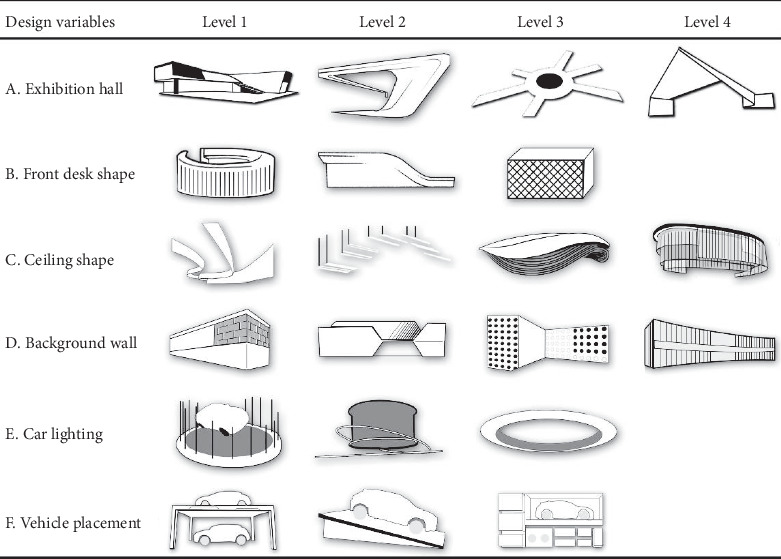
Form deconstruction chart.

**Figure 5:**
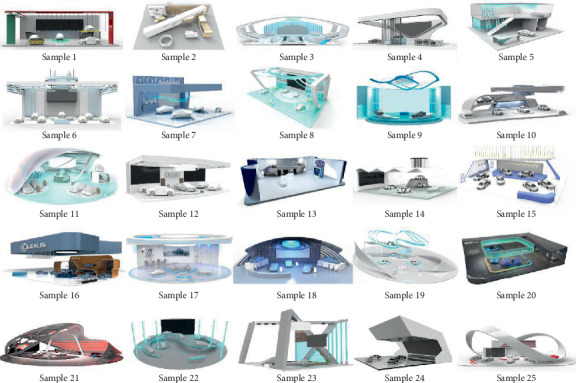
25 models of the car booth design.

The “fashionable” upper Kansei factor corresponds to six median design variables: *A*, the overall shape of the exhibition hall; *B*, the front desk shape; *C*, the ceiling shape; *D*, the background wall; *E*, the car lighting method; and F, the vehicle placement method. Among them, there are 4^3^ × 3^3^ = 1728 kinds of automobile booth design combinations produced by 21 lower design levels (Figure 4) of six design variables. In order to avoid stereotypes or brand factors affecting the experimental results, this article will construct a new automobile booth design as a questionnaire test sample. It would take a lot of time and effort to generate so many solutions. Therefore, this paper borrows the orthogonal design in the SPSS software to obtain the optimal combination of modeling parameters. Orthogonal design is a partial factor design. Through this program, the product portfolio can be reduced to a certain range that can be processed, while maintaining orthogonality. After executing the program, 25 representative solutions are obtained to cover various design variables and levels. Graduate students of industrial design from Nanchang University used the 3D software to generate 25 booth design models (Figure 5). The 100 experts that are mentioned above were invited to evaluate 25 models of car display design models with a 7-point Likert scale. A score of one indicates a low degree of fashion, a score of seven indicates a high degree, and the middle is a transition score. “Fashionable” is the Kansei evaluation index. The specific results are shown in Table 5.

### 3.3. Using NN to Establish the Mapping Model

This paper uses a three-layer neural network with a single hidden layer. The input layer has 21 nodes, that is, six morphological variables with a total of 21 levels. The input data are 25 design models. One node in the output layer is the “fashionable” perceptual evaluation. The number of nodes in the hidden layer generally uses the following formula:

(7) $$\begin{array}{c} m=\frac{n+l}{2}, \end{array}$$

where *m* is the number of nodes in the hidden layer, *n* is the number of nodes in the input layer, and *l* is in the output layer. Shen and Wang [43] found that when equation (7) was used to calculate the number of nodes in the hidden layer, the results were more accurate. Therefore, the number of nodes in the hidden layer is determined as (21 + 1)/2 = 11.

Because design variables cannot be directly used as input parameters, specific design elements of the automobile display design need to be coded. The number of bits encoded in each sample is the same as the total number of design levels, which is 21. Each design level code has only one number as 1, and the rest are 0. For example, in the orthogonal experiment, the type of sample 1 in the *A*–*F* design level is 4, 1, 2, 1, 3, and 1, respectively, then the code can be converted to 000110001001000001100, and the codes of other samples can be obtained in the same way as input layer parameters. In addition, since the output parameters of the training function need to be in the interval [0,1], while the Kansei evaluation results are obviously not completely in this interval, the Kansei evaluation values need to be normalized. This paper uses the fast linear transformation algorithm of equation (1), and after normalizing the data, it is in a suitable interval. Then, it can be imported as an output parameter into the NN model of the automobile booth design for training.

In this study, the first 20 samples are set as the training set, and the last five samples as the test set. Newff is used to create the NN. The activation function of the hidden layer uses a logarithmic sigmoid transfer function:

(8) $$\begin{array}{c} f\left(x\right)=\frac{1}{1+e^{-x}}\left(0≺f\left(x\right)≺1\right). \end{array}$$

**Figure 6:**
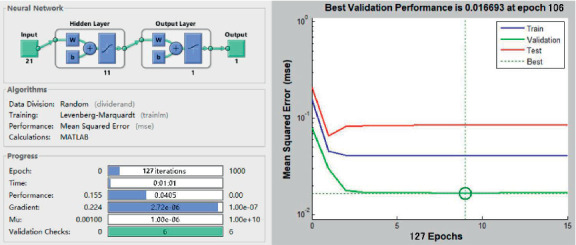
The performance of NN.

**Figure 7:**
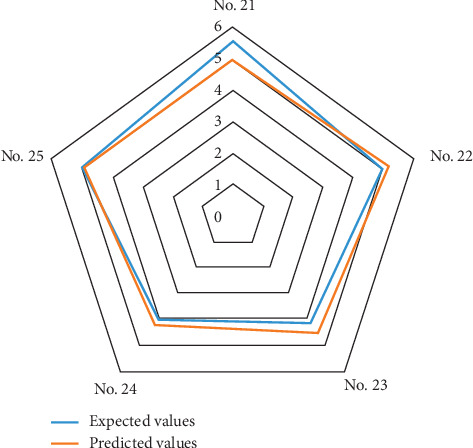
The radar map for validation.

The output layer uses a linear function purelin, the training function uses Trainlm, the number of learning is set to 10,000, and the error is 10^−4^. When the training reaches 127 iterations, the training purpose is achieved and the training is stopped. So far, the NN model of the automobile booth design has been obtained (Figure 6). In order to verify the performance of the NN model, the five samples in the test set are encoded as input layer parameters, and then the output layer values are obtained. At the same time, the predicted value and the measured value are tested by RMSE. The method of calculating standard deviation is used to evaluate the performance of a model, and the root of mean square error (RMSE) is commonly used and is given as

(9) $$\begin{array}{c} \text{RMSE}=\sqrt{\frac{\sum_{i=1}^{n}{\left(x_{i}-x_{0}\right)}^{2}}{n}}, \end{array}$$

where *x*_*i*_ is the *i*^th^ output value predicted by the model and *x*_0_ is the expected values assessed by the subjects in the experiment. If there is no difference or error between the output value and the expected value, the RMSE is 0. The normalized predicted value is compared with the measured value to obtain the RMSE (Table 6). Only when the RMSE of the NN is small, it means that the established NN architecture can be used for prediction, judgment, and inference.  Figure 7 is drawn to show a fitting diagram showing the relationship between the predicted value and the actual value of the output of the NN model. This result shows that the model has good consistency.

**Figure 8:**
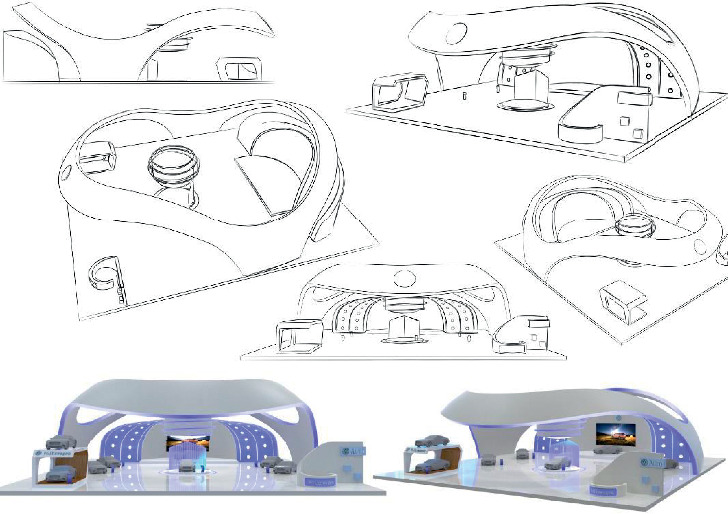
Novel design of the car booth.

Based on the trained NN model, 1728 kinds of morphological combinations of the automobile booth design are imported into the computer as input layer parameters, and the Kansei evaluation values that are corresponding to each combination are calculated. The output layer parameter codes of the top three items with the highest “fashionable” perceptual evaluation value after operation are 233233 (Kansei value: 4.4704), 213233 (Kansei value: 4.4619), and 333231 (Kansei value: 4.4614), and the corresponding best design element combinations are 010000100100100001001, 010010000100100001001, and 001000100100100001100. According to the form combination corresponding to the highest perceptual evaluation value, the design of the automobile booth design is as shown in Figure 8. Finally, the abovementioned 100 expert groups are invited again to implement a seven-point Likert scale on the design plan, and a “fashionable” Kansei evaluation value of 6.238 was obtained, which is significantly higher than the average of 3.5 points, so it illustrates the attractive product developed by the proposed combination model design has met customer preferences.

## 4. Analysis and Discussion

Compared with traditional Miryoku engineering methods, combining GRA and NN can achieve the weight ranking of upper Kansei factors and establish a nonlinear mapping function between upper and lower specific conditions, which solves the problem that Miryoku engineering can only provide customer preferences and the qualitative relationships between design elements. This paper executes the operation process of Miryoku engineering first to obtain 16 higher-level abstract Kansei factors, which express the user's glamour and emotional experience on the car display design. In order to distinguish the importance of these emotional elements, GRA was used to replace the simple operations of the previous sum of mentions, Kano model, or AHP method. The weight of the Kansei index was measured using the concept of grey correlation. It is found that the top three factors include fashionable (0.878), attractive (0.871), and noble (0.865) and the last three factors include retro (0.509), warm (0.628), and fantasy (0.667). This result shows that users attach the greatest importance to the innovative form of the automobile exhibition booth, and the fashionable product shape is still the soul of the design. Secondly, the noble and attractive booth design is easy to attract users' attention, and designers should give priority to these users' emotional preferences. On the contrary, the retro style of the booth is the least likely to be loved by users. At the same time, designers can ignore the warm and fantasy emotional images under the premise of limited resources.

With the rapid development of the global economy, car manufacturers have gradually attached more importance to the sales process than the development of the performance of the car itself. Various forms of exhibitions, sales, annual meetings and other exhibitions are in line with the needs of the entire social and economic development. This paper evaluates the charismatic characteristics of the automobile booth design in the market and investigates the quantitative relationship between the customer's abstract Kansei factor and booth design elements. Finally, the combination of the best perceptual evaluation was selected from more than a thousand design form combinations, that is, the combination of *A*2 + *B*3 + *C*3 + *D*2 + *E*3 + *F*3, and based on this, the most innovative automobile booth can be designed. In this paper, GRA and NN in artificial intelligence technology are used to accurately calculate the mapping function between the weight of the upper abstract sensation and the corresponding lower specific design conditions in the Miryoku engineering process. Therefore, designers can focus on the attractive factors of customer preferences obtained from the research results and formulate new product development strategies in a planned way to shorten the time of research and improve customer satisfaction.

## 5. Conclusion

In the fierce global auto sales market, an excellent automobile booth design can distinguish its own products from many homogeneous competitions, attract more visitors to stay, and increase product sales. The main purpose of this study is to develop an attractive automobile booth design combined with GRA and NN on the evaluation structure map platform established by Miryoku engineering. First, Miryoku engineering obtained 16 representative upper-level Kansei factors, 20 original reasons, and 77 lower-level specific design conditions based on a survey of 12 experts' group. Secondly, in artificial intelligence technology, GRA is used to replace the traditional frequency summing method, and the key Kansei factors are obtained by using the calculation method of the grey relationship degree. In the end, NN was used to establish a mapping relationship between the key Kansei factors and the corresponding design elements of the automobile booth shape and explore the optimal design to enhance the attractiveness of the automobile booth layout.

The limitation of this research is as follows. First of all, although the hierarchical structure of expert opinion, which is extracted by Miryoku engineering, is detailed and exquisite, it may be difficult to comprehensively record the subjective emotions by using only adjectives. Secondly, NN is always criticized for its black-box property, and other artificial intelligence technologies such as rough set theory and support vector machine can be applied in the future. After these, people's preference factors are different because of different genders, education levels, and occupations, so studies can be grouped based on demographic variables. Last, this paper only considers the morphological changes of the booth design, and the future research scope should be expanded to over booth size, finish materials, lighting, and signage.

## Figures and Tables

**Figure 9:**
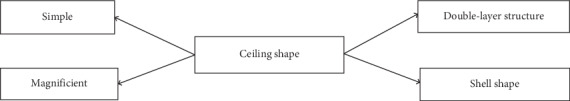
A single evaluation project construction diagram.

**Table 1:** A brief comparison between this paper and the previous ones.

Literature	Expert interview	Kansei evaluation	Function evaluation	Filter product design elements	Synthesis
This paper	EGM	GRA			NN
Wu and Cheng [16]	EGM	Fuzzy Kano, fuzzy AHP			QT-I
Wu and Kang [23]	EGM	Fuzzy AHP			
Kang et al. [5]	EGM	Fuzzy Kano, fuzzy AHP			Fuzzy QFD
Kang et al. [8]	EGM				QT-I
Kang et al. [24]	Focus group			Fuzzy Delphi	FWARM
Chen and Li [10]	EGM				QT-I
Shen [13]	EGM				QT-I
Zhang et al. [15]	EGM			ANP	
Wang and Hsueh [25]			AHP, Kano model		DEMATEL
Wang [26]			AHP, TRIZ		QFD
Wang [27]	Focus group		TRIZ, FCPR		RST

*Notes*. FWARM: fuzzy weighted association rule mining; TRIZ: theory of inventive problem solving; DEMATEL: decision-making trial and evaluation laboratory; RST: rough set theory; FCPR: fuzzy cognitive pairwise rating.

**Table 2:** 16 representative Kansei words.

Warm	Wide	Simple	Noble	Dynamic	Technological	Industrial	Retro
Youthful	Attractive	Passionate	Fantasy	Absorbed	Fashionable	Interesting	Exquisite

**Table 3:** Kansei evaluation values.

Kansei factors	1	2	3	4	5	6	7	8	9	10	11	12	13	14
Warm	3.25	3.45	3.16	5.63	4.47	3.75	3.59	3.90	3.98	3.90	3.92	4.43	3.98	4.12
Wide	5.47	5.31	5.76	5.29	5.63	4.37	5.04	5.31	5.24	5.31	4.86	4.67	5.61	5.12
Simple	5.59	5.29	5.61	4.94	5.35	4.51	5.31	5.33	5.31	4.98	4.71	4.98	5.49	5.31
Noble	5.20	3.98	5.24	4.96	5.18	4.71	4.98	5.10	4.71	5.02	4.24	5.06	4.80	4.35
Dynamic	4.86	3.76	5.94	3.53	4.51	5.71	5.45	4.98	4.53	5.61	4.16	5.06	4.96	4.39
Technological	5.69	4.25	5.76	3.75	4.88	5.35	5.43	4.49	4.73	5.65	4.37	5.29	5.37	4.75
Industrial	5.14	4.84	5.16	3.53	4.51	5.00	5.22	3.71	4.71	4.94	4.90	4.51	5.00	4.69
Retro	2.33	2.82	2.75	3.59	3.35	3.33	3.29	4.02	3.43	3.39	3.61	3.31	3.37	3.18
Youthful	5.33	4.73	5.35	4.69	4.78	5.20	5.29	4.24	4.61	5.35	4.16	5.10	5.12	4.63
Attractive	5.08	4.29	5.51	4.82	4.94	5.18	5.22	4.73	4.55	5.27	4.29	5.16	4.98	4.51
Passionate	4.80	3.96	5.61	3.96	4.22	5.25	5.39	4.86	4.35	4.92	4.00	4.76	4.88	4.18
Fantasy	3.16	2.90	3.43	4.35	4.80	4.25	3.76	3.96	3.59	4.73	3.27	4.84	4.10	3.65
Absorbed	3.86	3.49	4.41	4.53	4.57	4.53	4.49	4.94	4.33	5.00	4.12	4.86	4.53	4.35
Fashionable	4.75	4.12	5.10	4.65	4.69	5.45	5.18	4.49	4.10	5.20	4.18	4.92	4.80	4.39
Interesting	3.96	3.47	4.29	3.94	3.96	4.88	4.92	4.00	3.80	4.76	3.90	4.22	4.20	3.80
Exquisite	5.00	4.04	4.71	5.04	4.86	5.02	5.04	3.92	4.61	5.2	4.16	4.90	4.90	4.39

**Table 4:** Ranking of GRD.

Kansei factors	GRD	Ranking
Fashionable	0.878	1
Attractive	0.871	2
Noble	0.865	3
Youthful	0.847	4
Industrial	0.797	5
Dynamic	0.790	6
Absorbed	0.787	7
Passionate	0.780	8
Technological	0.762	9
Interesting	0.726	10
Simple	0.707	11
Wide	0.705	12
Fantasy	0.667	13
Warm	0.628	14
Retro	0.509	15

**Table 5:** Experiment layout.

*U*	*A*	*B*	*C*	*D*	*E*	*F*	Value
1	1	3	2	4	1	1	3.75
2	1	1	1	3	3	3	4.91
3	4	3	3	1	1	3	5.03
4	4	2	4	3	1	1	4.49
5	1	1	1	1	1	1	4.55
6	3	2	2	1	2	3	4.06
7	1	2	4	1	2	2	4.63
8	2	2	1	2	1	3	4.56
9	4	1	1	4	2	2	4.45
10	1	3	1	2	2	1	4.74
11	2	1	3	3	2	1	5.28
12	2	2	1	4	1	2	3.74
13	3	1	1	1	1	2	4.39
14	4	2	1	1	2	1	4.44
15	1	2	2	3	1	2	3.53
16	4	1	2	2	3	2	4.46
17	3	1	4	2	1	1	4.19
18	3	3	1	3	2	2	4.40
19	2	1	2	1	2	1	5.39
20	1	1	4	4	2	3	4.64
21	1	1	3	1	1	2	4.73
22	1	2	1	1	3	1	4.93
23	2	3	4	1	3	2	4.58
24	3	2	3	4	3	1	4.79
25	1	2	3	2	2	2	5.03

**Table 6:** NN model verification.

	No. 21	No. 22	No. 23	No. 24	No. 25	RMSE
Expected values	4.73	4.93	4.58	4.79	5.03	0.3134
Predicted values	4.4724	5.0519	4.6342	4.8727	4.9427	

## Data Availability

The experiment data used to support the findings of this study are included in the article.

## Appendix

Specific questionnaire in EGM:

(1) The specific questioning method in Section 3.1 is stated as follows:
Q1: “Do you prefer sample 10 or sample 15?”
    Answer: “I prefer sample 15”
Q2: “Why do you prefer sample 15?”
    Answer: “Because of the ceiling shape of sample 15!”
    After determining the median original reason preferred by the subject, continue to question the upper abstract reason of ladder up and the specific details of its composition ladder down.
(1) Ladder up: continue to ask high-level abstract reasons for “ceiling shapes.”
Q3: Why do you like the “ceiling shape” of sample 15?”
    Answer: Because it looks “simple and magnificent”
(2) Ladder down: based on the “ceiling shape”, continue to ask the testers about the specific design elements that constitute a “simple and atmospheric ceiling shape” and analyze the physical properties of the automobile booth that the testers love.
Q4: What design factors make you feel the simplicity of the ceiling?
    Answer: “Double-layer structure” and “Shell shape”
    From the above questioning method, a three-level structure diagram of the upper abstract reason for the evaluation item, the median original evaluation item, and the lower specific conditions is organized (Figure 9).
